# *Streptococcus pneumoniae* and *Haemophilus* species colonization in health care workers: the launch of invasive infections?

**DOI:** 10.1186/s13104-016-1877-x

**Published:** 2016-02-04

**Authors:** Supram Hosuru Subramanya, Sangita Thapa, Sanjiv Kumar Dwedi, Shishir Gokhale, Brijesh Sathian, Niranjan Nayak, Indira Bairy

**Affiliations:** Department of Microbiology, Manipal College of Medical Sciences, Pokhara, Nepal; Department of Community Medicine, Manipal College of Medical Sciences, Pokhara, Nepal; Department of Microbiology, Melaka Manipal Medical College, Manipal University, Manipal, India

**Keywords:** Health care workers, Colonization, *Haemophilus influenzae*, *S. pneumoniae*

## Abstract

**Background:**

*Streptococcus pneumoniae* and *Haemophilus influenzae* are important human pathogens. The risk of airborne and droplet-transmitted respiratory tract infections in healthcare workers (HCW) is substantial. The aim of this study was to determine the extent of oropharyngeal colonization with *S. pneumoniae* and *Haemophilus* spp. their antibiogram and risk factors of colonization in HCW at a tertiary care center, Western Nepal.

**Methods:**

During 3 month period, 100 oropharyngeal swab specimens were collected from HCW of Manipal Teaching Hospital and 50 from non HCW from community. All the 150 specimens were screened for *Haemophilus* spp. and *S. pneumoniae* by standard techniques. Serotyping of *H. influenzae* type b was done by using specific antiserum. Antibiotic sensitivity patterns of isolates were determined by modified Kirby Bauer disc diffusion method. Association between the groups was analyzed using the Pearson χ^2^ test and Fisher exact test. A forward step logistic regression model was used to identify significant predictors for colonization.

**Result:**

Sixty-five percent of HCW were colonized with *S. pneumoniae* and/or *Haemophilus* species compared to 32 % of non-HCW. Health care workers had odd ratio (OR) 3.946 [CI (1.916, 8.128)] times more tendency of colonization compared to non-HCW (P < 0.05). Pneumococcal colonization was observed high among smokers (81.5 %). Amongst HCW, post graduate resident doctors had higher rate of colonization (83.3 %) followed by interns (64.9 %), least being amongst the laboratory workers (58.3 %).

**Conclusion:**

The higher rate of colonization amongst HCW raises the possibility of occupational risk as well as horizontal spread of infections.

## Background

*Haemophilus influenzae* and *Streptococcus pneumoniae* are important bacterial pathogens, which can cause invasive diseases and respiratory infections in susceptible individuals. The clinical infection is preceded by asymptomatic colonization of the human pharynx [1]. Pharyngeal carrier rate of *H. influenzae* and *S. pneumoniae* varies globally. Humans are the only known asymptomatic carriers/reservoir [2]. Pharyngeal carriage of potential pathogens is important as it is both the major source of horizontal spread of this pathogen within the community and the prerequisite of invasive disease.

Transmission of *H. influenzae* or *S. pneumoniae* occurs through direct contact with respiratory droplets from pharyngeal carrier or a patient, or indirectly through contamination via fomites, although firm evidence for this mechanism is lacking [3, 4]. The period of communicability for pneumococcal and haemophilus disease is unknown, but it is plausible that transmission can occur as long as the organism appears in respiratory secretions [4]. Increased risk for disease among close contacts of patients with non-b or non-typeable *H. influenzae* has not been identified [5]. Unimmunized children younger than 4 years of age and older adults and patients with sickle cell disease, asplenia, HIV, certain immunodeficiency syndromes, and malignant neoplasms are at increased risk for invasive Haemophilus and Pneumococcal disease [6].

Close proximity of persons together with handling of human secretions make health care workers particularly vulnerable to transmission of droplet-transmitted infections. To the best of our knowledge there is limited data on the oropharyngeal carrier state of *S. pneumoniae* and *Haemophilus* species in healthy health care workers. Therefore this study was conducted to determine the rate of carrier state of these pathogens in HCW who have constant exposure to wide varieties of patients and to determine the associated risk factors for colonization.

## Methods

### Study population

HCW from various clinical departments and diagnostic laboratories of Manipal Teaching Hospital, Pokhara, Nepal, who often have contact with patients and/or handling of clinical specimens were enrolled in this study. Study was conducted department wise, including all grades of HCW (consultants, in-charges, nurses, post graduates medical students and interns). The healthy non-HCW like basic science faculty and adult volunteers from community who have no contact with health care settings were enrolled in the study. The age of the participants ranged between 20 and 72 years.

### Period of study

The study was carried out from March to July 2014.

### Sample size calculation

In a pilot study for power 85 % and α error 5 % with 95 % level of confidence showed proportion of colonization in health care workers group to be 0.60 and it was 0.30 in non-health care workers group. We estimated total sample size of 49 in each group.

### Specimen processing

**Fig. 1:**
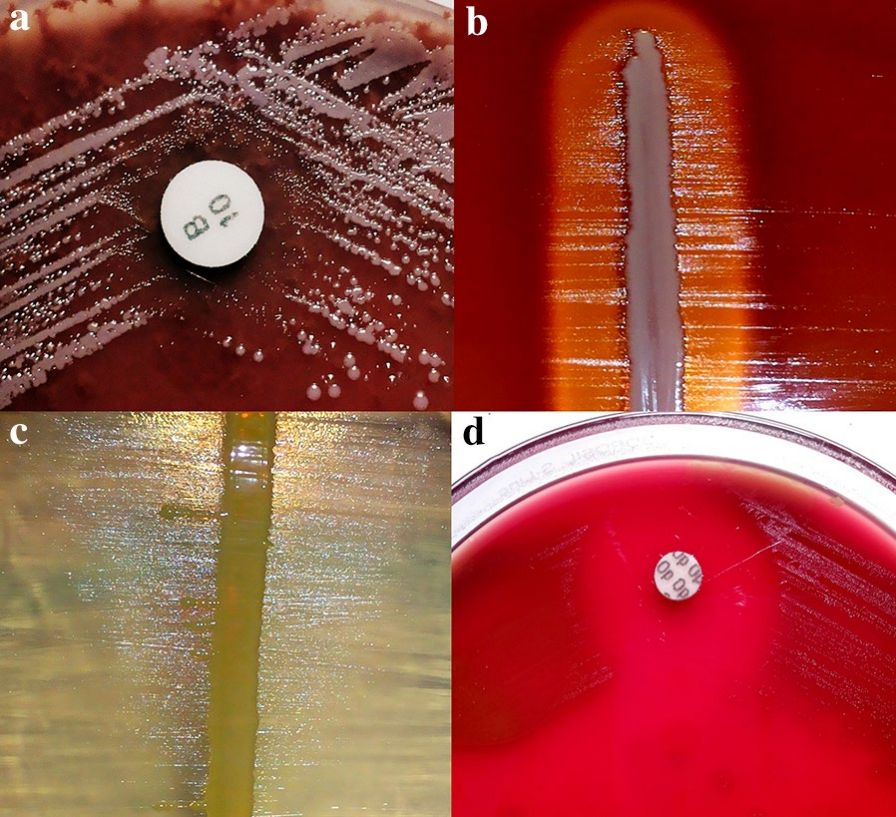
Representative panels showing identification techniques for the organisms. **a** Chocolate agar inoculated with the throat swab specimen showing Pinpoint, shiny colonies surrounding bacitracin disc (10 units) suggestive of *Haemophilus* spp. **b**, **c** Satellitism demonstrated in blood agar (*H. influenzae*) and nutrient agar (*Haemophilus* spp.) respectively. **d** Optochin sensitivity for *S. pneumoniae*

Selected volunteers were examined for any evidence of upper respiratory tract infection. The findings were recorded as per the protocol. Throat swab specimens were collected from the volunteers who did not have any clinical evidence of infection. The oropharyngeal swab specimens were collected from 100 HCW of Manipal Teaching hospital and 50 non HCW from community and screened for *Haemophilus* spp. and *S. pneumoniae* by standard techniques [7]. Although the statistical derivation mentioned above provided initial estimation of the sample size to be 49 in each group, we opted to increase the number of samples from the HCW, firstly because the HCW belonged to a diverse group (Table 2) of individuals and secondly because higher number would yield better statistical interpretation of data. Briefly, a semi quantitative culture technique was adopted to inoculate the specimens onto blood agar and chocolate agar with optochin disc (5 µg) and bacitracin disc (10 units) respectively (Fig. 1). We did not use any transport medium for the specimens as the laboratory facility was nearby. However, all the specimens were sent to the laboratory and processed with minimum delay. Plates were incubated at 37 °C overnight in presence of 5–10 % CO_2_ and were examined after 24 h. Suspected alpha hemolytic colonies from blood agar were sub-cultured to obtain pure growth and processed for *S. pneumoniae* by Gram’s stain and optochin sensitivity testing and by bile solubility test. The translucent colony grown around the disc of bacitracin on chocolate agar (Fig. 1) was presumed as *Haemophilus* spp. and identified by Gram’s stain and confirmed by satellitism test. These were serotyped as *H. influenzae* type b by specific antiserum. Serotyping of *S. pneumoniae* was not performed. Antibiotic sensitivity pattern of isolates was determined by using modified Kirby-Bauer disc diffusion method as per CLSI guidelines [8]. Data on potential risk factors were gathered by confidential interview based on a standardized questionnaire.

**Table 1:** Colonization of organisms amongst HCW and non-HCW

Sl. No	Organism	Colonization overall (%), n = 150	Colonization HCW (%), n = 100	Colonization non-HCW (%), n = 50
1	*Heamophilus influenzae*	19 (12.67)	15 (15)	4 (8)
2	*Streptococcus pneumoniae*	27 (18)	21 (21)	6 (12)
3	*Heamophilus influenzae* + *Streptococcus pneumoniae*	6 (4)	5 (5)	1 (2)
4	*Heamophilus* spp. + *Streptococcus pneumoniae*	5 (3.33)	4 (4)	1 (2)
5	*Heamophilus* spp.	24 (16)	20 (20)	4 (8)

**Table 2:** Statistical analysis of various risk factors for colonization with *S. pneumoniae* and *Haemophilus* spp.

Characteristics	No. (%), N = 150	Colonized, n = 81 (%)	Not colonized, n = 69 (%)	Chi square, P value*	Odd ratio
*Occupation*					
HCW	100 (66.67)	65 (65)	35 (35)	0.001	3.946
Non-HCW	50 (33.3)	16 (32)	34 (68)		1
*Gender*					
Female	73 (48.7)	36 (49.3)	37 (50.7)	0.262	–
Male	77 (51.3)	45 (58.4)	32 (41.6)		
*Smoking*					
Non-smoker	73 (48.7)	38 (52.1)	35 (47.9)	0.642	–
Smoker	77 (51.3)	43 (55.8)	34 (44.2)		
*Designation*					
Consultants	18 (12.0)	10 (58.8)	08 (41.2)	0.002	–
Residents	18 (12.0)	15 (83.3)	03 (16.7)		
Interns	37 (24.7)	24 (64.9)	13 (35.1)		
Nursing staffs	15 (10.0)	09 (60.0)	06 (40.0)		
Laboratory staffs	12 (8.0)	07 (58.3)	05 (41.7)		
Basic science faculties and community subjects	50 (33.3)	16 (32.0)	34 (68.0)		
*History of recurrent RTI*					
No	127 (84.7)	68 (53.5)	59 (46.5)	0.792	–
Yes	23 (15.3)	13 (16.0)	10 (43.5)		
*Oropharyngeal examination*					
Normal	137 (91.3)	71 (51.8)	66 (48.2)	0.083	–
Enlarged or inflamed tonsils	13 (8.7)	10 (76.9)	03 (23.1)		
*Close contact with children*					
No	90 (60.0)	54 (60.0)	36 (40.0)	0.071	–
Yes	60 (40.0)	27 (45.0)	33 (47.8)		
*History of antibiotic use in preceding 15 days*					
No	145 (96.7)	78 (53.8)	67 (46.2)	0.784	–
Yes	05 (3.3)	03 (46.2)	02 (40.0)		
*History of Pneumococcal and Haemophilus disease*					
No	150 (100)	81 (54.0)	69 (46.0)	–	–
Yes	00 (00)	00 (00)	00 (00)		
*Vaccination status*					
No	150 (100)	81 (54.0)	69 (46.0)	–	–
Yes	00 (00)	00 (00)	00 (00)		

* P values <0.05 were considered statistically significant

### Statistical analysis

Relation between groups was analyzed using the Pearson χ^2^ test and Fisher exact test. A forward step logistic regression model was used to identify significant predictors for colonization. P values <0.05 were considered statistically significant. The analyses were performed using the IBM SPSS Statistics 20 software from IBM Corporation, Armonk, New York, USA.

### Ethical clearance

Ethical committee approval was taken from the institutional ethical committee, Manipal Teaching hospital, Pokhara, Nepal. The Research was conducted in accordance to latest version of the Declaration of Helsinki.

### Consent

Written informed consent from the participant was obtained before enrolling in the study.

## Result

**Fig. 2:**
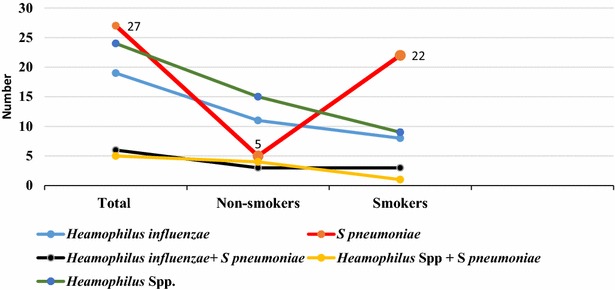
Comparison of oropharyngeal colonization amongst smokers and nonsmokers

Out of a total of 150 healthy volunteers, 81 (54 %) were colonized with *S. pneumoniae* and/or *Haemophilus* species. Colonization of *S. pneumoniae* and *Haemophilus* spp. amongst HCW and non-HCW are summarized in Table 1. Several potential risk factors for colonization were analyzed. Out of 100 HC volunteers sixty-five (65 %) were colonized with *S. pneumoniae* and/or *Haemophilus* spp. compared to 16 (32 %) of non-HCW. HCW were having OR 3.946 [CI (1.916, 8.128)] times more tendency of colonization compared to non-HCW (P < 0.05). Out of the total 25 *H. influenzae* isolates, 3 (12 %) were *H. influenzae* type b (Hib) and interestingly all these 3 isolates were found to be the oropharyngeal colonizers in HCW. There was significant association between smoking and bacterial colonization (P = 0.013). *S. pneumoniae* colonization was higher in smokers (81.5 %). Figure 2 depicts the pattern and number of oropharyngeal colonization amongst both smokers and nonsmokers. Amongst HCW, residents had higher rate of colonization (83.3 %) followed by interns (64.9 %), lowest rate of colonization being amongst the laboratory workers (58.3 %). A forward step logistic regression model was used to analyze the following potential risk factors: gender, recurrent upper respiratory tract infection, history of antibiotic intake, previous history of *S. pneumoniae* and *Haemophilus* disease, close contact with children, designation, throat examination findings and vaccination status. All the above mentioned risk factors were not associated with colonization by *S. pneumoniae* and/or *Haemophilu*s spp. Personal details of the volunteers and the risk factors for colonization with *S. pneumoniae* and *Haemophilus* spp. are summarized in Table 2.

**Fig. 3:**
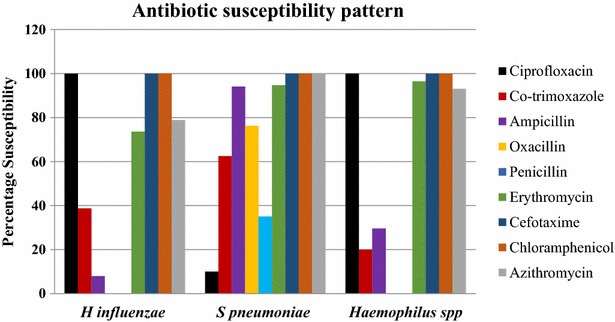
Antibiotic susceptibility pattern of the isolates depicting 100 % susceptibility of all three species towards cefotaxime and chloramphenicol. Whereas all *Haemophilus* species were susceptible to ciprofloxacin, only 10 % of *S. pneumoniae* were susceptible to this drug. It is noteworthy to observe that more than 70 % of *Haemophilus* species were resistant to ampicillin

On analyzing the antibiotic susceptibility pattern of the isolates, it was noted that all *H. influenzae* and *Haemophilus* spp. were susceptible to ciprofloxacin, cefotaxime and chloramphenicol. Only 8 % of the *H. influenzae* isolates and 29.6 % of the *Haemophilus* spp. were susceptible to ampicillin. Susceptibility towards co-trimoxazole amonogst *Haemophillus* species was not encouraging as well (20–38.8 %). Amongst the *S. pneumoniae* isolates 90–100 % were susceptible to ampicillin, erythromycin, cefotaxime, chloramphenicol and azithromycin. However the percentage susceptibility towards penicillin, ciprofloxacin, oxacillin and co-trimoxazole were found to be 35.1, 10, 76.7 and 62.5 respectively (Fig. 3).

## Discussion

Oropharynx colonization by encapsulated organisms like *S. pneumoniae* or *Haemophilus* spp. is very frequent, especially in children, and the spectrum of serotypes varies globally [9]. To our knowledge, this is the first study of oropharyngeal colonization with *S. pneumoniae* and *Haemophilus* spp. in healthy health care workers in Nepal. The interesting findings were that 65 % of HCW were colonized either by *S. pneumoniae* or *Haemophilus* spp. compared to 35 % of the non-HCW. We guess the isolation rates of *S. pneumoniae* and *Haemophilus* spp. projected by us by direct plating method would not have been any better had we utilized enrichment culture technique [10]. Notwithstanding the above, nasopharyngeal swab collection from study subjects might have provided better yield of organisms [11]. In this study, health care exposure was related to increased risk for colonization. These findings are noteworthy because *S. pneumoniae* or *Haemophilus* spp. like many other bacterial pathogens can be transmitted from patients to HCWs and vice versa [12, 13]. However, the information about the role and extent of such transmission is scanty in the literature. Besides, there is very little awareness regarding respiratory bacterial infections as an occupational health risk for HCWs.

The importance of *Haemophilus* species other than *H. influenzae* in human infections has been increasingly recognized in recent years [14]. *Haemophilus parainfluenzae* and other non-*H. influenzae* species, although human commensals, are infrequently reported to be pathogenic [15]. Difficulty in speciating *Heamophilus* and differentiating the other species from *H. influenzae*, in the past, might have led to misdiagnosis of infections caused by these organisms [16]. Thus, the presence on the respiratory mucosa, of non-*H. influenzae* species may serve as a source of genetic material for horizontal gene transfer to *H. influenzae* and thus might provide an easy access for *H. inflenzae* in acquiring new genes from the available gene pool maintained by other *Haemophilus* species [17].

Cigarette smoking is the strongest independent risk factor for invasive pneumococcal disease among immunocompetent, nonelderly adults [18]. The high rate of pneumococcal colonization in smokers in this study supports the previous study results [2, 19, 20]. Reducing smoke exposure may reduce pneumococcal carriage [20]. Camilli et al., reported that Pneumococcal vaccination increases *H. influenzae* nasopharyngeal carriage in children [21]. Other factors like upper respiratory infections, antibiotic intake, close contact with children are known risk factors for colonization of these organisms in pharynx [22, 23], but the small number of isolates in this study are insufficient to draw such a conclusion.

During the past few decades, antibiotic-resistant *Haemophilus* spp. and *S. pneumoniae* strains have appeared, and the major resistance mechanism proposed, was production of beta-lactamase. Unlike the aforementioned  studies in which beta lactamase production and MIC determination amongst the isolates were highlighted, our study lacked these data. However our in vitro results documented that there was a high rate of drug resistant *Haemophilus* spp. and *S. pneumoniae* colonization in our study subjects (Fig. 3). Knowledge of antibiotic resistance patterns of colonizers, with the potential to invade is an important instrument in establishing reference guidelines for the management of acute respiratory tract infections [24], as well as to develop the prevention strategies for pneumococcal or *Heamophilus* diseases.

In Nepal, large-scale Hib or Pneumococcal vaccination programs are rare, and their effect has not yet been evaluated sufficiently. Earlier studies showed that in Hib vaccinated population, the prevalence of airway carriage was significantly decreased [25]. However, the conjugated vaccine protects only partially against the carrier status and does not completely eliminate the risk of infection by this microorganism [25–27]. Since man is the only reservoir and colonization has a relevant role in the transmission cycle of invasive disease caused by this microorganism, it may be useful to vaccinate the risk group like HCW and to monitor the impact of such a measure. However,  because of limited data, additional studies are needed to adequately assess the impact of vaccine introduction on carriage of respiratory bacteria in this country.

## Conclusion

This study divulges the high carriage rate of* Haemophilus* species and *S. pneumoniae* in HCW compared to non-HCW. The higher rate of colonization amongst HCW raises the possibility of occupational risk as well as horizontal spread of infections. Thus a large scale study involving serotyping and further genotyping would provide adequate information for better understanding the clinical significance and molecular epidemiology of these organisms.
